# Associations between pregnancy loss and common mental disorders in women: a large prospective cohort study

**DOI:** 10.3389/fpsyt.2024.1326894

**Published:** 2024-03-08

**Authors:** Qiaoqiao Shen, Wenfang Zhong, Xiaomeng Wang, Qi Fu, Chen Mao

**Affiliations:** ^1^ School of Public Health, Southern Medical University, Guangzhou, Guangdong, China; ^2^ School of Nursing, Southern Medical University, Guangzhou, Guangdong, China

**Keywords:** cohort study, mental health, miscarriage, pregnancy termination, stillbirth

## Abstract

**Background:**

Increasing evidence suggests that pregnancy loss can lead to negative emotional outcomes, such as anxiety and depression, for women. However, limited knowledge exists regarding the long-term risk of mental disorders among individuals who have experienced pregnancy loss.

**Objective:**

To investigate the associations between pregnancy loss and the risk of common mental disorders.

**Methods:**

In the UK Biobank, a total of 218,990 women without any mental disorder at baseline were enrolled between 2006 and 2010 and followed until October 2022. Information on the history of pregnancy loss was obtained through self-reported questionnaires at baseline. Cox proportional hazard regression models were used to estimate adjusted hazard ratios (HRs) and 95% confidence intervals (CIs) for associations between pregnancy loss and common mental disorders.

**Results:**

During a median follow-up time of 13.36 years, there were 26,930 incident cases of common mental disorders. Incidence rates of common mental disorders were elevated among women with a history of stillbirth (HR 1.15, 95% CI: 1.07–1.23), miscarriage (HR 1.06, 95% CI: 1.02–1.10), or pregnancy termination (HR 1.21, 95% CI: 1.17–1.25) compared to those without such experiences. Furthermore, the risk of common mental disorders significantly increased in women with two or more miscarriages (HR 1.14, 95% CI: 1.08–1.19) or two or more pregnancy terminations (HR 1.39, 95% CI: 1.30–1.48).

**Conclusions:**

Pregnancy loss is associated with an increased risk of common mental disorders in women later in life. These findings may contribute to the enhancement of long-term monitoring and prevention of common mental disorders for women with such a history.

## Introduction

Pregnancy loss refers to the death of an unborn baby (fetus) at any stage during pregnancy, mainly including stillbirth, miscarriage, and pregnancy termination (1). Globally, more than 20% of pregnancies end in miscarriage (2), and approximately 2.7 million stillbirths occur annually (3). Moreover, an estimated 73 million pregnancies are terminated each year, affecting a substantial proportion of women with unintended pregnancies (4).

Any form of pregnancy loss, whether unexpected, such as miscarriage or stillbirth, or elective, such as termination, constitutes one of the most distressing events in a woman’s life (5). Previous studies indicate that fetal death can elicit profound emotional responses in women, leaving them feeling grief-stricken, shocked, helpless, and guilty (6, 7). In recent years, increased attention has been directed toward the stigma and societal silence surrounding pregnancy loss (8). Many women express difficulty in discussing their experiences of losing a baby with family members or healthcare professionals (9, 10). This complicates the grieving process, intensifying feelings of isolation and potentially leading to long-term psychological consequences (8–10). A recent study by Herbert et al. (11) revealed significantly elevated rates of depression and anxiety among women who experienced pregnancy loss.

While a clear relationship exists between pregnancy loss and adverse mental health outcomes, existing studies have certain limitations in their designs. For example, extensive qualitative research has been dedicated to interpreting psychological outcomes after pregnancy loss (6, 7, 9, 10). Although these studies provide detailed insights into the experiences of bereaved women, they cannot effectively measure the extent and type of mental health outcomes (12). Furthermore, most quantitative studies have employed a cross-sectional design (11), and the few prospective studies were conducted with limited participants (11) or short-interval follow-ups (13, 14). Consequently, there is limited knowledge about the long-term risk of mental disorders among women who have experienced pregnancy loss.

The present study utilized a large prospective cohort from the UK Biobank to evaluate whether pregnancy loss is associated with an increased risk of long-term mental disorders, including substance use disorders, mood (affective) disorders, and anxiety and stress-related disorders.

## Materials and methods

### Study population

The UK Biobank is a large prospective cohort study established to investigate the environmental exposure and genetic predisposition associated with a wide range of diseases (15). Between 2006 and 2010, participants aged 40 to 69 were recruited from 22 centers in England, Wales, and Scotland. After providing informed consent, participants completed health status and medical history questionnaires, underwent physical and functional assessments, and submitted biological samples. Throughout the study, all disease events, drug prescriptions, and participant deaths were documented in a database, utilizing the centralized UK National Health Service (15). In this study, female participants with incomplete pregnancy loss data (n = 6,387), those with mental disorders at baseline (n = 47,350), and individuals who subsequently withdrew from the study (n = 571) were excluded from the analysis. A total of 218,990 female participants (80.1%) were included in the final cohort (see **Supplementary Figure S1**).

### Ascertainment of the exposure

The history of stillbirth, miscarriage, and pregnancy termination was assessed through a self-reported questionnaire at baseline. Participants responded to the following queries: “Have you ever had any stillbirths, spontaneous miscarriages or terminations?” If affirmative, they provided information on the number of stillbirths, miscarriages, and terminations. Due to an insufficient number of events for further analysis, stillbirths, miscarriages, and pregnancy terminations were categorized into two groups (0 and ≥1), three groups (0, 1, and ≥2), and three groups (0, 1, and ≥2), respectively.

### Ascertainment of covariates

Sociodemographic variables included age, ethnicity (white or other), education (with or without a college/university degree), and stressful life events such as illness, injury, bereavement, divorce, and financial difficulties (yes or no), as well as the Townsend deprivation index. Health and lifestyle factors encompassed body mass index, sleep duration (≤ 6h, 7–8 h, or ≥ 9 h per night), overall health (poor, fair, good, or excellent), hypertension (yes or no), and diabetes (yes or no). Reproductive factors included the number of live births (0, 1, 2, or ≥3), history of oral contraceptive pill use (ever or never), and history of hormone replacement therapy use (ever or never). Detailed information on these measurements is available online through the UK Biobank showcase (www.ukbiobank.ac.uk).

### Ascertainment of the outcomes

Mental disorders, identified through self-report, primary care records, hospital admissions, or death registries, were classified according to the International Classification of Diseases coding system 10 (ICD-10). The date of diagnosis was defined as the earliest occurrence of a mental disorder code, regardless of the data source. This study focused on several outcomes: all common mental disorders (F10–F19, F30–F39, and F40–F48); substance use disorders (F10–F19), primarily encompassing alcohol-dependent or tobacco-related disorders; mood (affective) disorders, including depression (F30–F39); and anxiety and stress-related disorders (F40–F48). Participants with any of these outcomes before the deadline were coded as 1; otherwise, they were coded as 0. Follow-up time was calculated from the baseline date to the diagnosis of a mental disorder, death, or the end of follow-up on October 31, 2022, whichever occurred first.

### Statistical analyses

Baseline characteristics were presented as the number (percentage) for categorical variables and the mean (standard deviation, SD) for continuous variables. Missing values for covariates were imputed using Multivariate Imputation by Chained Equations with five iterations (16). Detailed information on the number of missing covariates is provided in **Supplementary Table S1**.

Cox proportional hazards models were employed to calculate hazard ratios (HRs) and 95% confidence intervals (CIs) for common mental disorders associated with pregnancy loss. The proportional hazard assumption was assessed using Schoenfeld residuals (17), and no violation of this assumption was observed in the analyses. Stratified analysis was conducted to estimate potential modification effects by age (< 60 or ≥ 60 years), ethnicity (White or others), education (with or without a college or university degree), Townsend deprivation index (≤ median or > median), obesity (yes, body mass index ≥ 30 kg/m^2^ or no, body mass index < 30 kg/m^2^), sleep duration (healthy sleep duration, 7–8 h, or unhealthy sleep duration, ≤ 6h or ≥ 9 h), stressful life events (yes or no), number of live births (0 or ≥1), oral contraceptive pill use (ever or never), and hormone replacement therapy use (ever or never). An interaction term between the exposure of interest and the subgroup was fitted to obtain the p-value.

Sensitivity analyses were performed to test the robustness of the results. First, considering the potential impact of pregnancy on women’s mental health, participants without a history of pregnancy were excluded in a sensitivity analysis. Second, to avoid potential reverse causation, participants who developed common mental disorders within the first 2 years of follow-up were excluded. Additionally, the analysis was confined to participants with complete covariate data, minimizing any potential bias from missing information.

Statistical analyses were conducted using R software version 4.1.3 (R Development Core Team, Vienna, Austria). P values < 0.05 (two-sided) were considered statistically significant.

### Ethics approval

The UK Biobank study received approval from the North West Multi-Centre Research Ethics Committee. All participants provided electronic informed consent during recruitment. This study was conducted under UK Biobank permission (UKB application 43795).

## Results

### Characteristics of the study participants

In this study, a total of 218,990 women were included, with an average age of 56.52 years (SD, 8.02 years). The majority of participants (92.0%) were White, and approximately one-third (32.4%) held a college/university degree. More than 80% of participants reported using oral contraceptive pills, while a minority (18.5%) had no history of live births. The incidence rates for stillbirth, miscarriage, and pregnancy termination were 2.5%, 20.6%, and 13.5%, respectively. **Table 1** presents the baseline characteristics of women with and without a history of pregnancy loss.

**Table 1:** Baseline characteristics between women with and without a history of stillbirth, miscarriage, or pregnancy termination.

Characteristic	Overall	Stillbirth		Miscarriage		Pregnancy termination	
		Never	Ever	Never	Ever	Never	Ever
**Sample size**	218,990	213,493	5,497	173,825	45,165	189,331	29,659
**Age, years**	56.52 (8.02)	56.46 (8.02)	58.83 (7.58)	56.59 (8.00)	56.23 (8.09)	56.87 (7.97)	54.24 (7.96)
Ethnicity							
White	201,393 (92.0)	196,758 (92.2)	4,635 (84.3)	160,153 (92.1)	41,240 (91.3)	175,487 (92.7)	25,906 (87.3)
Others	17,597 (8.0)	16,735 (7.8)	862 (15.7)	13,672 (7.9)	3,925 (8.7)	13,844 (7.3)	3,753 (12.7)
Education							
With a college/university degree	70,849 (32.4)	69,575 (32.6)	1,274 (23.2)	55,664 (32.0)	15,185 (33.6)	59,766 (31.6)	11,083 (37.4)
Without a college/university degree	148,141 (67.6)	143,918 (67.4)	4,223 (76.8)	118,161 (68.0)	29,980 (66.4)	129,565 (68.4)	18,576 (62.6)
**Townsend deprivation index**	-1.44 (2.99)	-1.45 (2.98)	-0.86 (3.36)	-1.43 (2.99)	-1.47 (2.99)	-1.54 (2.93)	-0.78 (3.26)
**Body mass index, kg/m^2^**	26.93 (5.08)	26.91 (5.08)	27.90 (5.33)	26.90 (5.07)	27.06 (5.14)	26.94 (5.07)	26.90 (5.16)
Sleep duration, hours							
7–8	151,483 (69.2)	147,992 (69.3)	3,491 (63.5)	120,530 (69.3)	30,953 (68.5)	131,336 (69.4)	20,147 (67.9)
≤ 6	51,872 (23.7)	50,345 (23.6)	1,527 (27.8)	40,732 (23.4)	11,140 (24.7)	44,264 (23.4)	7,608 (25.7)
≥ 9	15,635 (7.1)	15,156 (7.1)	479 (8.7)	12,563 (7.2)	3,072 (6.8)	13,731 (7.3)	1,904 (6.4)
Stressful life events							
Yes	96,982 (44.3)	94,311 (44.2)	2,671 (48.6)	75,610 (43.5)	21,372 (47.3)	81,967 (43.3)	15,015 (50.6)
No	122,008 (55.7)	119,182 (55.8)	2,826 (51.4)	98,215 (56.5)	23,793 (52.7)	107,364 (56.7)	14,644 (49.4)
Overall health							
Excellent	41,029 (18.7)	40,317 (18.9)	712 (13.0)	32,943 (19.0)	8,086 (17.9)	35,720 (18.9)	5,309 (17.9)
Fair	38,220 (17.5)	36,858 (17.3)	1,362 (24.8)	29,927 (17.2)	8,293 (18.4)	32,550 (17.2)	5,670 (19.1)
Good	133,474 (60.9)	130,348 (61.1)	3,126 (56.9)	106,259 (61.1)	27,215 (60.3)	115,823 (61.2)	17,651 (59.5)
Poor	6,267 (2.9)	5,970 (2.8)	297 (5.4)	4,696 (2.7)	1,571 (3.5)	5,238 (2.8)	1,029 (3.5)
Hypertension							
Yes	104,452 (47.7)	101,270 (47.4)	3,182 (57.9)	83,099 (47.8)	21,353 (47.3)	92,185 (48.7)	12,267 (41.4)
No	114,538 (52.3)	112,223 (52.6)	2,315 (42.1)	90,726 (52.2)	23,812 (52.7)	97,146 (51.3)	17,392 (58.6)
Diabetes							
Yes	7,428 (3.4)	7,061 (3.3)	367 (6.7)	5,756 (3.3)	1,672 (3.7)	6,532 (3.5)	896 (3.0)
No	211,562 (96.6)	206,432 (96.7)	5,130 (93.3)	168,069 (96.7)	43,493 (96.3)	182,799 (96.5)	28,763 (97.0)
Number of live births							
0	40,616 (18.5)	40,367 (18.9)	249 (4.5)	37,556 (21.6)	3,060 (6.8)	35,312 (18.7)	5,304 (17.9)
1	28,240 (12.9)	27,509 (12.9)	731 (13.3)	22,224 (12.8)	6,016 (13.3)	23,055 (12.2)	5,185 (17.5)
2	97,154 (44.4)	94,850 (44.4)	2,304 (41.9)	75,817 (43.6)	21,337 (47.2)	85,185 (45.0)	11,969 (40.4)
≥ 3	52,980 (24.2)	50,767 (23.8)	2,213 (40.3)	38,228 (22.0)	14,752 (32.7)	45,779 (24.2)	7,201 (24.3)
Oral contraceptive pill use							
Ever	176,529 (80.6)	172,450 (80.8)	4,079 (74.2)	139,022 (80.0)	37,507 (83.0)	150,144 (79.3)	26,385 (89.0)
Never	42,461 (19.4)	41,043 (19.2)	1,418 (25.8)	34,803 (20.0)	7,658 (17.0)	39,187 (20.7)	3,274 (11.0)
Hormone replacement therapy use							
Ever	80,998 (37.0)	78,622 (36.8)	2,376 (43.2)	64,136 (36.9)	16,862 (37.3)	71,259 (37.6)	9,739 (32.8)
Never	137,992 (63.0)	134,871 (63.2)	3,121 (56.8)	109,689 (63.1)	28,303 (62.7)	118,072 (62.4)	19,920 (67.2)

Note. Data are described as the mean (standard deviation) or number (percentage).

### Associations of pregnancy loss with common mental disorders

During the median follow-up time of 13.36 years, 26,930 incident cases of common mental disorders were recorded. Anxiety and stress-related disorders were the most common (n = 12,986 [5.9%]), followed by mood (affective) disorders (n = 10,530 [4.8%]) and substance use disorders (n = 9,047 [4.1%]). The detailed distribution among the different categories of common mental disorders is shown in **Supplementary Table S2**.

Compared to women without such experiences, those with a history of one or more stillbirths showed an increased risk of common mental disorders (HR 1.15, 95% CI: 1.07–1.23). Similarly, women with one or more miscarriages had elevated risks (for 1 miscarriage, HR 1.06, 95% CI: 1.02–1.10; for ≥2 miscarriages, HR 1.14, 95% CI: 1.08–1.19), as did women with one or more pregnancy terminations (for 1 termination, HR 1.21, 95% CI: 1.17–1.25; for ≥2 terminations, HR 1.39, 95% CI: 1.30–1.48) for common mental disorders. Similar trends were observed for the various subtypes of common mental disorders associated with stillbirth, miscarriage, and pregnancy termination, as detailed in **Table 2**.

**Table 2:** Associations of stillbirth, miscarriage, and pregnancy termination with common mental disorders.

Outcome	Number of stillbirths		Number of miscarriages			Number of pregnancy terminations		
	0	1+	0	1	2+	0	1	2+
**All common mental disorders**	1.00 (reference)	1.15 (1.07, 1.23)	1.00 (reference)	1.06 (1.02, 1.10)	1.14 (1.08, 1.19)	1.00 (reference)	1.21 (1.17, 1.25)	1.39 (1.30, 1.48)
**Substance use disorders**	1.00 (reference)	1.19 (1.06, 1.33)	1.00 (reference)	1.10 (1.04, 1.17)	1.20 (1.11, 1.30)	1.00 (reference)	1.42 (1.34, 1.51)	1.80 (1.63, 1.98)
**Mood (affective) disorders**	1.00 (reference)	1.19 (1.08, 1.33)	1.00 (reference)	1.05 (1.01, 1.11)	1.21 (1.12, 1.30)	1.00 (reference)	1.17 (1.11, 1.24)	1.26 (1.13, 1.40)
**Anxiety and stress-related disorders**	1.00 (reference)	1.12 (1.01, 1.23)	1.00 (reference)	1.05 (1.00, 1.10)	1.10 (1.03, 1.18)	1.00 (reference)	1.16 (1.10, 1.22)	1.32 (1.20, 1.46)

Note. Data presented as hazard ratio (95% CI). The models were adjusted for age (continuous), ethnicity (White or others), education (with or without a college or university degree), Townsend deprivation index (continuous), body mass index (continuous), overall health (excellent, good, fair, or poor), hypertension (yes or no), diabetes (yes or no), sleep duration (≤ 6h, 7–8 h, or ≥ 9 h), stressful life events (yes or no), number of live births (0, 1, 2, or ≥ 3), oral contraceptive pill use (ever or never), and hormone replacement therapy use (ever or never).

### Subgroup and sensitivity analyses

**Figure 1:**
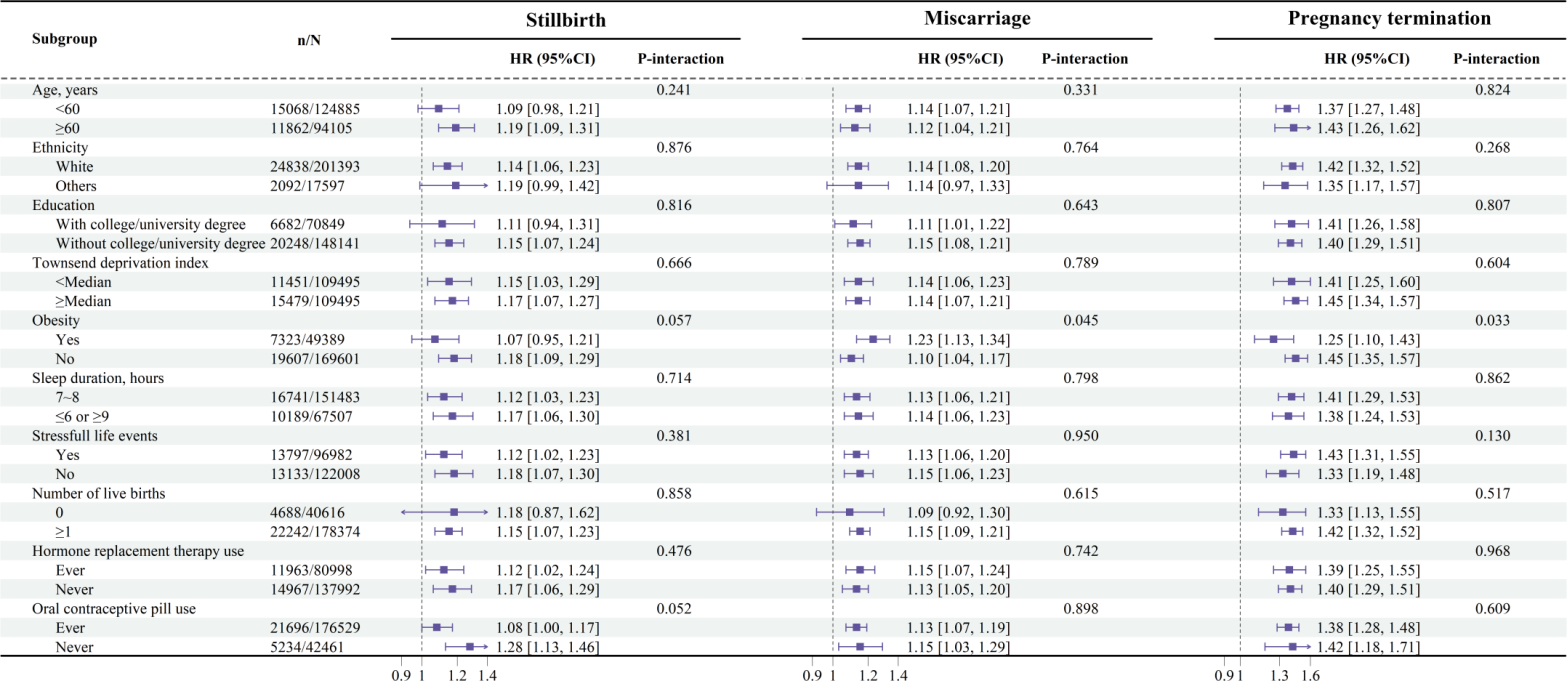
Associations of stillbirth, miscarriage, and pregnancy termination with common mental disorders stratified by potential risk factors. Results were adjusted for age, ethnicity (White or others), education (with or without a college or university degree), Townsend deprivation index, body mass index, overall health (excellent, good, fair, or poor), hypertension (yes or no), diabetes (yes or no), sleep duration (≤ 6h, 7–8 h, or ≥ 9 h), stressful life events (yes or no), number of live births (0, 1, 2, or ≥ 3), oral contraceptive pill use (ever or never), and hormone replacement therapy use (ever or never).

Subgroup analyses were conducted to assess the impact of potential confounding factors, as illustrated in **Figure 1**. Among participants with obesity, stronger associations between miscarriage, pregnancy termination, and common mental disorders were observed (P for interaction = 0.045 and 0.033, respectively). Furthermore, sensitivity analyses were performed to ensure the robustness of these findings. No statistically significant alterations were observed when excluding individuals who had never experienced pregnancy (see **Supplementary Table S3**), those who developed common mental disorders within the first 2 years of follow-up (see **Supplementary Table S4**), or those with missing covariate data (see **Supplementary Table S5**).

## Discussion

To our knowledge, this is the first cohort study investigating the associations between pregnancy loss and the long-term risk of common mental disorders. Our findings indicate that women with a history of stillbirth, miscarriage, or pregnancy termination are at an elevated risk of developing common mental disorders in later life. Subgroup and sensitivity analyses further supported the robustness of these findings.

Epidemiological research has consistently revealed a strong association between pregnancy loss in women and an increased incidence of short-term anxiety and depression (11, 18, 19). Within 1-2 months following a pregnancy loss, 18-32% of women experience anxiety disorders, while 11-30% exhibit depressive symptoms, as reported in numerous studies (18, 19). In a prospective cohort study, Farren et al. (14) observed a gradual decline in the prevalence of mental disorders among women following early pregnancy loss over time. However, at 9 months post-pregnancy loss, 17% exhibited moderate to severe anxiety, and 6% showed moderate to severe depression (14). Similarly, Lewkowitz and colleagues reported an increased risk of seeking medical care for mental illness among women within one year after stillbirth, with the highest risk occurring within four months postpartum (13). A systematic review revealed no significant differences in depression at 8 months and anxiety at 30 months following pregnancy loss between the stillbirth group and the control group (20). However, limitations in the included studies, primarily small sample sizes, and the exclusive reliance on screening questionnaires during longitudinal follow-up impeded a comprehensive and accurate assessment of the psychological trauma experienced by women following pregnancy loss (20). It is noteworthy that there is a scarcity of research based on large-scale medical diagnostic data to thoroughly investigate the long-term effects of pregnancy loss on the incidence of mental disorders in women beyond a 3-year timeframe. Consequently, there is an urgent need for high-quality, long-term follow-up studies to validate the current findings in this field.

Our study also identified an elevated risk of substance use disorders in women who have experienced pregnancy loss. Findings from Lewkowitz et al. (13) revealed a 2.41-fold heightened risk of emergency room visits or hospitalization within one year of stillbirth due to drug or alcohol use or dependence, in contrast to those with successful deliveries. A cohort study focusing on young women aged 18 to 23 demonstrated a heightened risk of tobacco dependence and illicit drug use in those with a history of miscarriage or abortion, compared to those who have never been pregnant (21). The experience of pregnancy loss can induce mental trauma in women, and when conventional coping mechanisms prove ineffective, some may turn to substances such as alcohol and tobacco for temporary stress relief (13, 21). However, this misuse of substances may exacerbate mental stress, contributing to a decline in mental well-being (22).

We sought to understand the possible pathways underlying the associations between pregnancy loss and common mental disorders through subgroup analysis. Although these associations were generally consistent across most subgroups, stronger links between miscarriage, pregnancy termination, and common mental disorders were observed among women with obesity. Obesity is frequently linked to an unhealthy lifestyle, may lead to body image dissatisfaction and diminished self-esteem, contributing to mental stress (23). These factors may adversely affect the psychological adaptive capacity of women post-pregnancy loss, hindering their mental recovery process (24). Furthermore, a proposed hypothesis suggests that inflammatory pathways may mediate the connection between pregnancy loss, particularly recurrent pregnancy loss, and the development of mental disorders (25). Women with a history of pregnancy loss exhibit a pro-inflammatory state (26, 27), characterized by elevated levels of high-sensitivity C-reactive protein closely associated with the subsequent risk of mental disorders (28, 29). Further investigation is essential to comprehend the potential mechanisms underlying the onset of mental disorders following pregnancy loss.

The present study contributes to heightening public awareness regarding the prevalence of common mental disorders in middle-aged and elderly women who have undergone pregnancy loss, thereby advancing the comprehension of enduring mental health risks associated with such experiences. In the United Kingdom, the National Health Service recommends the development of tailored screening tools and pathways for women grappling with mental health issues following baby loss (30). Implementation of this recommendation would enable early identification of symptoms related to common mental disorders, facilitating targeted interventions to address the distinctive challenges faced by middle-aged and elderly women with a history of pregnancy loss. Additionally, we advocate for continuous follow-up of women who have experienced pregnancy loss, with timely measures implemented to offer essential support and treatment, preventing the escalation of mental health issues and alleviating the overall burden on individuals and the healthcare system.

## Strengths and limitations

Our study possesses several strengths, including a large sample size, detailed information on different types of pregnancy loss, and a prospective cohort study design with linkages to national registries that minimized loss to long-term follow-up. However, our study also has some limitations. Firstly, pregnancy loss information relies on self-reporting by women, introducing potential bias due to the sensitive and distressing nature of such disclosures. Nevertheless, it is noteworthy that reproductive history data collected by the UK Biobank are based on extensive survey purposes, significantly mitigating the likelihood of bias in data collection. Secondly, precise timing information regarding pregnancy loss is unavailable, necessitating the exclusion of all participants with mental disorders before the baseline reporting time. This procedure may introduce measurement errors and potentially underestimate the true strength of the associations observed. Thirdly, the UK Biobank cohort is a relatively healthy, demonstrating a “healthy volunteer bias,” which may limit the generalizability of these findings to the broader population. Finally, although we adjusted for major confounding factors, residual confounding from unknown or unmeasured factors still remains possible.

## Conclusions

Different types of pregnancy loss, including stillbirth, miscarriage, and pregnancy termination, are all positively associated with common mental disorders. This study underscores the importance of paying increased attention to women who have experienced pregnancy loss and advocates for the provision of comprehensive support to mitigate the occurrence of common mental disorders within this population. Further research is needed to elucidate the relationship between pregnancy loss and long-term mental health in women, as well as to understand the underlying mechanisms of these associations.

## Data availability statement

Publicly available datasets were analyzed in this study. This data can be found here: https://www.ukbiobank.ac.uk/.

## Ethics statement

The studies involving humans were approved by North West Multi-Centre Research Ethics Committee. The studies were conducted in accordance with the local legislation and institutional requirements. The participants provided their electronic informed consent to participate in this study.

## Author contributions

QS: Conceptualization, Data curation, Formal analysis, Investigation, Methodology, Software, Validation, Visualization, Writing – original draft, Writing – review & editing. WZ: Conceptualization, Data curation, Formal analysis, Investigation, Methodology, Software, Validation, Visualization, Writing – review & editing. XW: Investigation, Methodology, Validation, Writing – review & editing. QF: Writing – review & editing. CM: Data curation, Funding acquisition, Methodology, Project administration, Resources, Supervision, Writing – review & editing.
