# Supplementary material for: Associations between pregnancy loss and common mental disorders in women: a large prospective cohort study
Source: Front Psychiatry. 2024 Mar 8;15:1326894. doi: 10.3389/fpsyt.2024.1326894 (PMC10957736; doi:10.3389/fpsyt.2024.1326894)
Supplement: Supplementary file 1 [file DataSheet_1.docx]

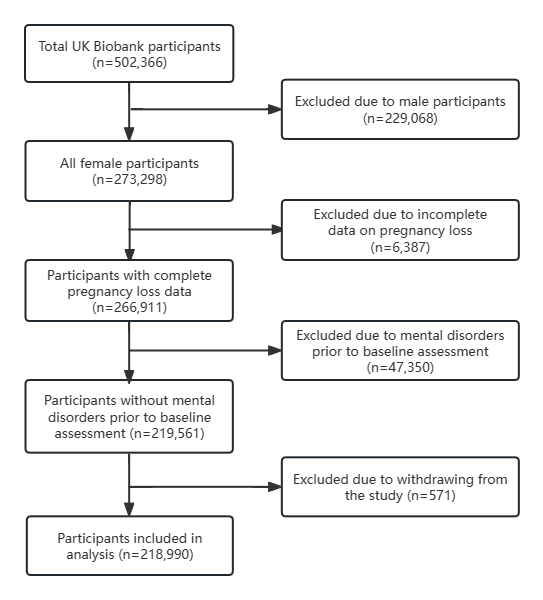


Figure S1. Flow diagram for study participants

Table S1. The numbers (percentages) of participants with missing covariates

| Covariate | n | % |
| --- | --- | --- |
| Ethnicity | 564 | 0.26 |
| Education | 3,974 | 1.81 |
| Townsend deprivation index | 239 | 0.11 |
| Sleep duration | 1,363 | 0.62 |
| Stressful life events | 3,146 | 1.44 |
| Body mass index | 945 | 0.43 |
| Overall health | 829 | 0.38 |
| Hormone replacement therapy use | 641 | 0.29 |
| Oral contraceptive pill use | 460 | 0.21 |
| Number of live births | 118 | 0.05 |
| Hypertension | 7 | 0.00 |

Table S2. Frequency distribution among the different categories of common mental disorders

| **Mental Disorder Description** | n | % |
| --- | --- | --- |
| **F10–F19 Substance use disorders** | 9,047 | 4.13 |
| [F10 mental and behavioral disorders due to use of alcohol](https://biobank.ndph.ox.ac.uk/showcase/field.cgi?id=130854) | 1,646 | 0.75 |
| [F11 mental and behavioral disorders due to use of opioids](https://biobank.ndph.ox.ac.uk/showcase/field.cgi?id=130856) | 34 | 0.02 |
| [F12 mental and behavioral disorders due to use of cannabinoids](https://biobank.ndph.ox.ac.uk/showcase/field.cgi?id=130858) | 45 | 0.02 |
| [F13 mental and behavioral disorders due to use of sedatives or hypnotics](https://biobank.ndph.ox.ac.uk/showcase/field.cgi?id=130860) | 18 | 0.01 |
| [F14 mental and behavioral disorders due to use of cocaine](https://biobank.ndph.ox.ac.uk/showcase/field.cgi?id=130862) | 8 | 0.00 |
| [F15 mental and behavioral disorders due to use of other stimulants, including caffeine](https://biobank.ndph.ox.ac.uk/showcase/field.cgi?id=130864) | 7 | 0.00 |
| [F16 mental and behavioral disorders due to use of hallucinogens](https://biobank.ndph.ox.ac.uk/showcase/field.cgi?id=130866) | 4 | 0.00 |
| [F17 mental and behavioral disorders due to use of tobacco](https://biobank.ndph.ox.ac.uk/showcase/field.cgi?id=130868) | 7,649 | 3.49 |
| [F18 mental and behavioral disorders due to use of volatile solvents](https://biobank.ndph.ox.ac.uk/showcase/field.cgi?id=130870) | 1 | 0.00 |
| [F19 mental and behavioral disorders due to multiple drug use and use of other psychoactive substances](https://biobank.ndph.ox.ac.uk/showcase/field.cgi?id=130872) | 18 | 0.01 |
| **F30–F39 Mood (affective) disorders** | 10,530 | 4.80 |
| [F30 manic episode](https://biobank.ndph.ox.ac.uk/showcase/field.cgi?id=130890) | 49 | 0.02 |
| [F31 bipolar affective disorder](https://biobank.ndph.ox.ac.uk/showcase/field.cgi?id=130892) | 181 | 0.08 |
| [F32 depressive episode](https://biobank.ndph.ox.ac.uk/showcase/field.cgi?id=130894) | 10,148 | 4.63 |
| [F33 recurrent depressive disorder](https://biobank.ndph.ox.ac.uk/showcase/field.cgi?id=130896) | 228 | 0.10 |
| [F34 persistent mood (affective) disorders](https://biobank.ndph.ox.ac.uk/showcase/field.cgi?id=130898) | 58 | 0.03 |
| [F38 other mood (affective) disorders](https://biobank.ndph.ox.ac.uk/showcase/field.cgi?id=130900) | 19 | 0.01 |
| [F39 unspecified mood (affective) disorder](https://biobank.ndph.ox.ac.uk/showcase/field.cgi?id=130902) | 131 | 0.06 |
| **F40–F48 Anxiety and stress-related disorders** | 12,986 | 5.93 |
| [F40 phobic anxiety disorders](https://biobank.ndph.ox.ac.uk/showcase/field.cgi?id=130904) | 1,413 | 0.65 |
| [F41 other anxiety disorders](https://biobank.ndph.ox.ac.uk/showcase/field.cgi?id=130906) | 10,029 | 4.58 |
| [F42 obsessive-compulsive disorder](https://biobank.ndph.ox.ac.uk/showcase/field.cgi?id=130908) | 75 | 0.03 |
| [F43 reaction to severe stress, and adjustment disorders](https://biobank.ndph.ox.ac.uk/showcase/field.cgi?id=130910) | 1,733 | 0.79 |
| [F44 dissociative (conversion) disorders](https://biobank.ndph.ox.ac.uk/showcase/field.cgi?id=130912) | 78 | 0.04 |
| [F45 somatoform disorders](https://biobank.ndph.ox.ac.uk/showcase/field.cgi?id=130914) | 508 | 0.23 |
| [F48 other neurotic disorders](https://biobank.ndph.ox.ac.uk/showcase/field.cgi?id=130916) | 48 | 0.02 |

Table S3. Associations of stillbirth, miscarriage, and pregnancy termination with common mental disorders after excluding participants without a history of pregnancy

| **Outcome** | **Number of stillbirths** | |  | **Number of miscarriages** | | |  | **Number of pregnancy terminations** | | |
| --- | --- | --- | --- | --- | --- | --- | --- | --- | --- | --- |
|  | **0** | **1+** |  | 0 | 1 | 2+ |  | 0 | 1 | 2+ |
| **All common mental disorders** | 1.00 (reference) | 1.14 (1.06, 1.22) |  | 1.00 (reference) | 1.05 (1.02, 1.09) | 1.13 (1.07, 1.18) |  | 1.00 (reference) | 1.21 (1.16, 1.25) | 1.39 (1.30, 1.48) |
| **Substance use disorders** | 1.00 (reference) | 1.17 (1.05, 1.31) |  | 1.00 (reference) | 1.07 (1.01, 1.14) | 1.16 (1.07, 1.26) |  | 1.00 (reference) | 1.39 (1.31, 1.48) | 1.77 (1.60, 1.95) |
| **Mood (affective) disorders** | 1.00 (reference) | 1.19 (1.07, 1.32) |  | 1.00 (reference) | 1.06 (1.00, 1.12) | 1.21 (1.13, 1.31) |  | 1.00 (reference) | 1.20 (1.13, 1.27) | 1.29 (1.16, 1.43) |
| **Anxiety and stress-related disorders** | 1.00 (reference) | 1.11 (1.00, 1.22) |  | 1.00 (reference) | 1.04 (0.99, 1.09) | 1.09 (1.02, 1.17) |  | 1.00 (reference) | 1.16 (1.10, 1.22) | 1.32 (1.19, 1.45) |

*Data presented as hazard ratio (95% CI). The models were adjusted for age (continuous), ethnicity (White or others), education (with or without a college or university degree), Townsend deprivation index (continuous), body mass index (continuous), overall health (excellent, good, fair, or poor), hypertension (yes or no), diabetes (yes or no), sleep duration (≤ 6h, 7–8 h, or ≥ 9 h), stressful life events (yes or no), number of live births (0, 1, 2, or ≥ 3), oral contraceptive pill use (ever or never), and hormone replacement therapy use (ever or never).

Table S4. Associations of stillbirth, miscarriage, and pregnancy termination with common mental disorders after excluding participants who developed common mental disorders within the first 2 years of follow-up

| **Outcome** | **Number of stillbirths** | |  | **Number of miscarriages** | | |  | **Number of pregnancy terminations** | | |
| --- | --- | --- | --- | --- | --- | --- | --- | --- | --- | --- |
|  | **0** | **1+** |  | 0 | 1 | 2+ |  | 0 | 1 | 2+ |
| **All common mental disorders** | 1.00 (reference) | 1.16 (1.08, 1.25) |  | 1.00 (reference) | 1.05 (1.02, 1.09) | 1.13 (1.07, 1.19) |  | 1.00 (reference) | 1.21 (1.17, 1.26) | 1.39 (1.29, 1.49) |
| **Substance use disorders** | 1.00 (reference) | 1.17 (1.04, 1.32) |  | 1.00 (reference) | 1.10 (1.04, 1.17) | 1.19 (1.09, 1.30) |  | 1.00 (reference) | 1.43 (1.34, 1.52) | 1.81 (1.63, 2.01) |
| **Mood (affective) disorders** | 1.00 (reference) | 1.22 (1.09, 1.36) |  | 1.00 (reference) | 1.05 (0.99, 1.11) | 1.20 (1.11, 1.30) |  | 1.00 (reference) | 1.19 (1.12, 1.26) | 1.23 (1.10, 1.38) |
| **Anxiety and stress-related disorders** | 1.00 (reference) | 1.14 (1.03, 1.27) |  | 1.00 (reference) | 1.04 (0.99, 1.10) | 1.10 (1.02, 1.19) |  | 1.00 (reference) | 1.16 (1.09, 1.22) | 1.35 (1.21, 1.49) |

*Data presented as hazard ratio (95% CI). The models were adjusted for age (continuous), ethnicity (White or others), education (with or without a college or university degree), Townsend deprivation index (continuous), body mass index (continuous), overall health (excellent, good, fair, or poor), hypertension (yes or no), diabetes (yes or no), sleep duration (≤ 6h, 7–8 h, or ≥ 9 h), stressful life events (yes or no), number of live births (0, 1, 2, or ≥ 3), oral contraceptive pill use (ever or never), and hormone replacement therapy use (ever or never).

Table S5. Associations of stillbirth, miscarriage, and pregnancy termination with common mental disorders after excluding participants with missing covariate data

| **Outcome** | **Number of stillbirths** | |  | **Number of miscarriages** | | |  | **Number of pregnancy terminations** | | |
| --- | --- | --- | --- | --- | --- | --- | --- | --- | --- | --- |
|  | **0** | **1+** |  | 0 | 1 | 2+ |  | 0 | 1 | 2+ |
| **All common mental disorders** | 1.00 (reference) | 1.14 (1.06, 1.22) |  | 1.00 (reference) | 1.06 (1.02, 1.10) | 1.13 (1.07, 1.19) |  | 1.00 (reference) | 1.21 (1.17, 1.26) | 1.40 (1.31, 1.49) |
| **Substance use disorders** | 1.00 (reference) | 1.17 (1.03, 1.31) |  | 1.00 (reference) | 1.10 (1.04, 1.17) | 1.18 (1.09, 1.29) |  | 1.00 (reference) | 1.44 (1.35, 1.52) | 1.82 (1.65, 2.01) |
| **Mood (affective) disorders** | 1.00 (reference) | 1.19 (1.06, 1.33) |  | 1.00 (reference) | 1.07 (1.01, 1.13) | 1.22 (1.13, 1.31) |  | 1.00 (reference) | 1.17 (1.10, 1.24) | 1.27 (1.14, 1.42) |
| **Anxiety and stress-related disorders** | 1.00 (reference) | 1.12 (1.01, 1.24) |  | 1.00 (reference) | 1.05 (0.99, 1.10) | 1.10 (1.02, 1.18) |  | 1.00 (reference) | 1.17 (1.11, 1.24) | 1.32 (1.20, 1.46) |

*Data presented as hazard ratio (95% CI). The models were adjusted for age (continuous), ethnicity (White or others), education (with or without a college or university degree), Townsend deprivation index (continuous), body mass index (continuous), overall health (excellent, good, fair, or poor), hypertension (yes or no), diabetes (yes or no), sleep duration (≤ 6h, 7–8 h, or ≥ 9 h), stressful life events (yes or no), number of live births (0, 1, 2, or ≥ 3), oral contraceptive pill use (ever or never), and hormone replacement therapy use (ever or never).
